# Brain activity during real-time walking and with walking interventions after stroke: a systematic review

**DOI:** 10.1186/s12984-020-00797-w

**Published:** 2021-01-15

**Authors:** Shannon B. Lim, Dennis R. Louie, Sue Peters, Teresa Liu-Ambrose, Lara A. Boyd, Janice J. Eng

**Affiliations:** 1 Graduate Studies in Rehabilitation Sciences, University of British Columbia, Vancouver, Canada; 2 Rehabiliation Research Program, GF Strong Rehabilitation Centre, 4255 Laurel St, Vancouver, BC V5Z 2G9 Canada; 3 Department of Physical Therapy, University of British Columbia, 212-2177 Wesbrook Mall, Vancouver, BC V6T 1Z3 Canada; 4 The Djavad Mowafaghian Centre for Brain Health, 212-2177 Wesbrook Mall, Vancouver, BC V6T 1Z3 Canada; 5 Centre for Hip Health and Mobility, Vancouver, Canada

**Keywords:** Stroke, Brain imaging, Gait, fNIRS, EEG, FDG-PET, Rehabilitation

## Abstract

Investigations of real-time brain activations during walking have become increasingly important to aid in recovery of walking after a stroke. Individual brain activation patterns can be a valuable biomarker of neuroplasticity during the rehabilitation process and can result in improved personalized medicine for rehabilitation. The purpose of this systematic review is to explore the brain activation characteristics during walking post-stroke by determining: (1) if different components of gait (i.e., initiation/acceleration, steady-state, complex) result in different brain activations, (2) whether brain activations differ from healthy individuals. Six databases were searched resulting in 22 studies. Initiation/acceleration showed bilateral activation in frontal areas; steady-state and complex walking showed broad activations with the majority exploring and finding increases in frontal regions and some studies also showing increases in parietal activation. Asymmetrical activations were often related to performance asymmetry and were more common in studies with slower gait speed. Hyperactivations and asymmetrical activations commonly decreased with walking interventions and as walking performance improved. Hyperactivations often persisted in individuals who had experienced severe strokes. Only a third of the studies included comparisons to a healthy group: individuals post-stroke employed greater brain activation compared to young adults, while comparisons to older adults were less clear and limited. Current literature suggests some indicators of walking recovery however future studies investigating more brain regions and comparisons with healthy age-matched adults are needed to further understand the effect of stroke on walking-related brain activation.

## Background

Stroke is a leading cause of adult long-term disability worldwide. The restoration of gait is rated as a high priority for stroke survivors [1, 2]. Yet, more than 50% of individuals living post-stroke do not independently walk within their community [3, 4]. Arguably, the efficacy of gait rehabilitation could be advanced with an individual’s personal brain activations [5, 6]. This notion of personalized medicine has become an important avenue of exploration and is now stated as a research priority within national funding agencies [7]. Determining neural correlates of walking is an important starting point in investigating how brain activation can be a valuable biomarker or indicator of neuroplasticity during the rehabilitation process.

Until recently, neural correlates of human walking were informed by studies with simulated or imagined walking tasks while under constrained brain imaging environments. The recent advancement in technologies such as portable electroencephalography (EEG), functional near-infrared spectroscopy (fNIRS), and radioactive tracing with positron emission topography (PET) or single-photon emission computerized tomography (SPECT) have allowed for investigation of brain function during real-time walking. Comparisons of simulated/imagined walking and real-time walking in healthy adults show many similarities in activation areas along the cortex, basal ganglia, brainstem, and cerebellum [8] and differences in motor preparatory areas (e.g., bilateral supplementary motor area (SMA)) and executive function areas (e.g., dorsolateral prefrontal cortex (PFC)) [9].

The ability to obtain measurements during real-time walking allows for investigation of brain activations associated with walking components that are necessary for successful community ambulation, such as acceleration/deceleration phases, steady-state walking, and complex situations that involve avoiding obstacles or doing multiple tasks at once (e.g., talking and walking). Previous studies show differing brain activities during these various components. In healthy adults, walking preparation increases PFC, premotor cortex (PMC), SMA and medial sensorimotor cortex (SMC) activity, whereas walking execution mainly activates SMA and medial SMC [10]. As the complexity of walking increases (e.g., walking while doing a secondary task), further increases of bilateral PFC activation are shown in healthy older adults [11]. When assessing brain activation during different components of walking within neurological populations, results are quite varied [12–14]. Other reviews investigating brain activation during real-time walking focus on the general neurological population category, rather than stroke specifically [8, 15]. Two systematic reviews exclusively looking at fNIRS studies in individuals with stroke only included three and five real-time walking studies [16, 17]. Their narrow inclusion criteria excluded some pertinent studies and more investigations have since been conducted in the stroke population. To facilitate rehabilitation of community ambulation post-stroke, a thorough understanding of how stroke affects functional brain activation during various walking components is necessary.

Thus, the purpose of the current systematic review is to consolidate work investigating the spatial and temporal brain activation of real-time walking in individuals with stroke. Specifically, studies will be described within three components:

1. Intention/acceleration: prior to walking onset or immediately post initiation of walking
2. Steady-state: during walking at a steady pace without additional tasks
3. Complex walking: walking with a secondary task or an externally cued gait

## Methods

The protocol for this systematic review was registered in the International Prospective Register of Systematic Reviews, PROSPERO (CRD42019127401, April 2019). This systematic review was conducted in accordance with PRISMA guidelines. A narrative synthesis of results was completed. If sufficient homogeneity in studies was present, quantitative pooling was planned (none was completed due to significant heterogeneity in methods).

### Search strategy and study selection

Six databases were used to search for studies published from inception to July 16, 2020: Medline (Ovid), Embase (Ovid), Pubmed, Web of Science, CINAHL (EBSCOHOST), and PsycInfo (EBSCOHOST). Search terms relating to Population (stroke), Intervention (real-time, upright walking), and Outcome (brain activation, fNIRS, EEG, PET or SPECT with radioactive tracing) were created for each database with keywords and medical subject headings (MeSH) terms as appropriate (Appendix Table 4).

Specific brain-imaging modalities were included in the search based on the ability to measure brain activation during real-time walking. In short, fNIRS takes advantage of the absorption properties of hemoglobin and utilizes near-infrared light to measure changes in regional (de)oxyhemoglobin concentration along the cortex (i.e., limited ability to measure subcortical structures). Similar to fMRI, fNIRS uses the theory of neurovascular coupling to infer real-time regional brain activity through changes in hemoglobin concentrations (i.e., more brain activation requires more oxygen and thus more oxyhemoglobin) [18]. EEG utilizes electrodes placed along specific points on the scalp and measures the net electrical activity across an ensemble of neurons within the cortical and subcortical layers with high temporal resolution. EEG is typically described through its frequency profile or an event-related potential, with increases in higher frequencies (e.g., beta band: 13–30 Hz) and larger baseline deflections indicating increased activation [19]. Finally, PET and SPECT scans utilize an injected tracer to assess metabolic uptake during the entire task or uptake period (i.e., not in real-time but representative of activation during the task). In most cases for PET, a fluorodeoxyglucose (FDG) tracer is used to follow the metabolic pathway of glucose (an excitatory neurotransmitter) and provides an indication of regions with increased excitatory neuronal activation [20].

Search results were imported into Covidence (Veritas Health Innovation, Australia) for duplicate removal and screening. Full-text reviews of the screened articles were then assessed for inclusion based on the criteria below. Reference lists of included full-texts and relevant reviews were hand-searched for additional articles. Screening of titles, abstracts, and full-text reviews were independently completed by two authors (SBL, DRL). Inconsistencies were discussed between reviewers; if a consensus was not reached, a third author (SP) was consulted.

### Inclusion and exclusion criteria

Articles were included if they assessed brain activity during real-time, upright gait in adults (> 18 years of age) post-stroke. Studies were also included if brain activity was assessed immediately prior to gait in order to assess the preparation and initiation component of gait. All types of study designs were considered for inclusion (i.e., case studies, pre-post studies, cross-sectional studies, randomized controlled trials). Published abstracts and conference abstracts were also included if adequate information regarding brain imaging methods and walking tasks were provided. Studies that included individuals of various neurological conditions were only included if at least 50% of the sample had a stroke. Due to the infancy of this field, inclusion of a broad range of study designs and mixed groups was deliberate to ensure that no relevant stroke findings were missed. Articles involving animal models, pediatric strokes (< 18 years of age), and studies published in languages other than English were not included.

### Data extraction

Data from the full-text articles were extracted independently by two authors (SBL, SP). The data extraction form included the following article details: title, year, author, journal, country of study, study type, participants (number, age, time since stroke, type of stroke, severity of stroke), technique used for measuring brain activity (type of device, density of recording, regions of interest, rigour of measuring brain activity), type of walking task (acceleration/initiation, steady-state, complex), walking trial (length of trial, number of trials, speed of walking), intervention (if applicable), comparator groups (no comparator, older adults, young adults, other neurological groups), and main findings. If the walking task was separated within the analysis, results from the first portion were placed in the acceleration/initiation category and the second portion were placed in the steady-state category. If distinct walking tasks were not explicitly investigated (e.g., acceleration/initiation versus steady-state) and a study investigated a single walking period including the acceleration phase, it was categorized as steady-state walking. Corresponding authors were contacted for further clarification and details on the studies as needed.

The National Institutes of Health (NIH) Study Quality Assessment Tools [21] were used to determine the quality of each study by two authors (SBL, DRL). This tool was designed based on quality assessment methods, concepts, and other tools developed by numerous national and international agencies. As indicated by the NIH Tools, separate assessments were completed based on the study type and an overall study rating of poor, fair, or good was provided by each assessor. According to the NIH descriptions, “a ‘good’ study has the least risk of bias, and result are considered to be valid. A ‘fair’ study is susceptible to some bias deemed not sufficient to invalidate its results. The fair quality category is likely to be broad, so studies with this rating will vary in their strengths and weaknesses. A ‘poor’ rating indicates significant risk of bias” [21]. Inconsistencies were discussed between assessors; if a consensus was not reached, a third author (SP) was consulted.

## Results

### Search yield

**Fig. 1:**
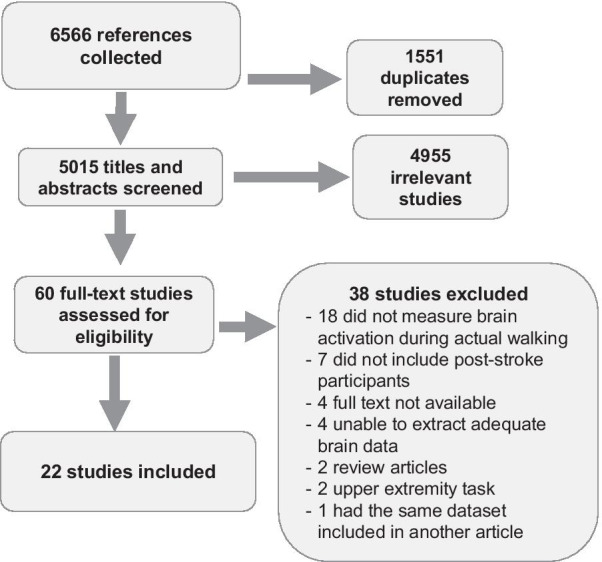
PRISMA flow chart

A total of 6566 articles were retrieved from the six databases. Once duplicates were removed and titles and abstracts were screened, 60 full-text articles were reviewed for inclusion. Thirty-eight articles were excluded (see Fig. 1 for details). Twenty-two articles met the inclusion criteria for this systematic review.

### Study characteristics

Articles were published between 2000 and 2020. Studies were conducted in Canada (n = 2), France (n = 1), Italy (n = 2), Japan (n = 6), Korea (n = 2), the Netherlands (n = 1), Spain (n = 1), Taiwan (n = 3), United Kingdom (n = 1), and USA (n = 3).

Eighteen studies were cross-sectional [22–39], two studies were randomized controlled trials [40, 41], and two studies were uncontrolled pre-post trials [42, 43]. One study was published as a book chapter [28] and two studies were published conference abstracts [30, 31]. Further detail on the book chapter [28] and conference abstract [31] were obtained through personal communication. Subsequent publication of the healthy older adult group [44] and a subsequent preprint currently under peer-review [45] were used to extract participant and task details.

### Stroke population

A total of 290 stroke participants (mean (SD): 59.0 (21.3) years, 24.6 (26.9) months post-stroke) were investigated in the 22 studies. All studies included stroke-only participant groups with no studies including participants with other neurological conditions. Studies included a range in number of participants, from 1 to 33 individuals with stroke. Specifically, the majority of studies (10 studies) had less than 10 participants, five had between 10 and 20 participants, six had between 20 and 30 participants, and only one study had greater than 30 participants. Six studies included individuals in the subacute stage of stroke (< 6 months post), 15 studies included chronic stroke (> 6 months), and one study did not report the post-stroke time. Fifteen studies included individuals who could walk independently, six studies were classified participants as having severe-moderate walking impairments or required maximum-moderate walking assistance, and one study did not report walking ability (Appendix Table 5).

Twelve studies provided details on individual lesion locations, eight studies only reported lesion side, stroke type (ischemic or hemorrhagic), or depth of lesion (cortical or subcortical), and three did not report any detail on the stroke. Overall, lesion locations were heterogeneous with only three studies being more specific in inclusion criteria: Mihara et al. [39] and Mori et al. [26] specifically excluded participants with lesions over recording areas—cortical lesions and PFC lesions, respectively—and Mitchell et al. [31] only included participants with lesions around the basal ganglia or internal capsule (Appendix Table 5). Two studies specifically reported observing no brain activation over lesioned areas [24, 43]. All other studies did not report accounting for lesion location in data analysis.

Three studies [35, 36, 46] made comparisons with a younger group of adults, and seven studies made comparisons with older or age-matched adults [22, 23, 25, 26, 31, 36, 39].

### Study quality

Eight studies were assessed as “good” [23, 29, 33, 36–38, 40], nine studies were “fair” [25, 26, 31, 34, 35, 39, 41–43], and five studies were “poor” [24, 27, 28, 30, 32] using the NIH Study Quality Assessment Tools (Appendix Table 6). In general, most studies neglected to report recruitment methods, number of eligible participants, or sample size justifications. Walking tasks were generally described with adequate detail. Most studies assess participants at their comfortable walking pace and walking tasks were similar between participants within each study. Methods of recording functional brain activation were described in good detail, though specific details on landmarking for device set up and localization of functional brain regions were often absent.

### Brain recording details

Three methods of measuring brain activity were used: EEG (n = 7), fNIRS (n = 14), and [18F]-FDG-PET (n = 1). Brain recording set-ups and regions of interest varied from investigating one brain region to whole head measures. The majority of studies used the 10/10 or 10/20 International system [47] to place their channels, four studies aligned their channels based on estimates from representative participants [24, 25, 39, 43], three reported a rough location of where channels were placed (e.g., high and lateral on forehead) [27, 33, 36], and two did not report how channels were placed [30, 32].

Results within the following sections will be described in the following order: activations in the stroke population, relationships between brain activation and performance, and brain activations in comparison to healthy individuals.

#### Brain activation during initiation and acceleration of walking

**Fig. 2:**
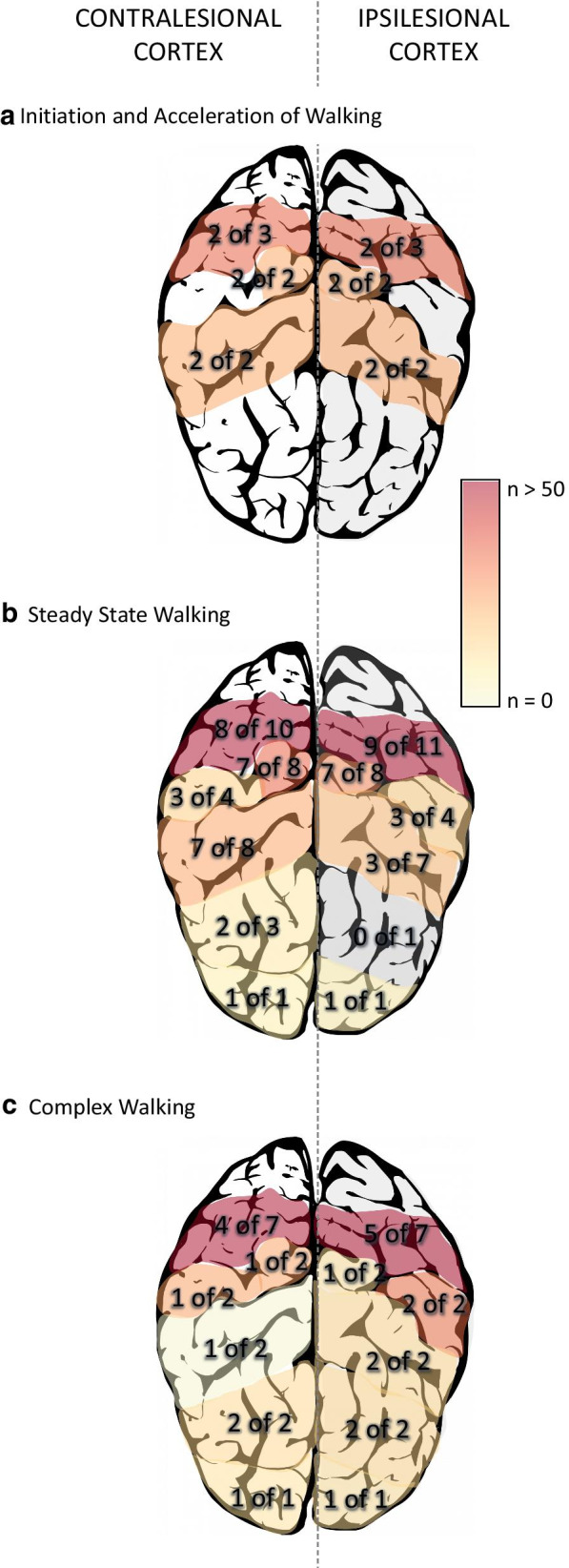
Summary of regional activations for the **a** initiation/acceleration phase of walking, **b** steady-state phase of walking, and **c** complex walking tasks. Numbers within the brain indicate how many studies showed increased activation out of the total number of studies that investigated the region. The colour gradient indicates the sum total of subjects within the studies showing increased activation

Three studies were included within this category [29, 36, 39] (Table 1). Overall, activations were bilateral with no differences between lesioned or non-lesioned hemispheres. For the stroke participants, two [36, 39] of the three studies showed increased activation in bilateral PFC, and both studies that looked at SMA and SMC showed increased activations with walking compared to standing [29, 36] (Fig. 2a).

**Table 1:** Studies investigating initiation and acceleration of walking listed by increasing walking speed

Authors	Device [ROI]; method of placing channels	Walking time analyzed, task and speed: m/s (SD)	Results
(Mihara et al., 2007) [39]	fNIRS [PFC, SMA, SMC]; anatomically guided from 2 representative subjects	4–10 s post-treadmill start, Treadmill, fast, comfortable pace: Stroke: 0.33 (0.22) Healthy: 0.97 (SD not provided)	1. Increased PFC, SMA, and SMC activation 2. Greater increase in right PFC and bilateral SMA for the stroke compared to healthy group 3. No between group differences for SMC
(Hawkins et al., 2018) [36]	fNIRS [PFC]; placed high and lateral on the forehead	7–37 s after start command, Overground, preferred speed: Stroke: 0.51 (0.27) Older adults: 1.07 (0.16) Young adults: 1.28 (0.18)	1. Increased PFC activity 2. No difference in PFC activity between hemispheres, side of stroke, or gender 2. Greater increase in PFC activity compared to young adults, but not compared to older adults
(Sburlea et al., 2015) [29]	EEG [Whole head]; 10/10 system	−1.5–0 s prior to start, Overground, comfortable pace: exact pace not specified	1. Activation at SMA and M1 500 ms prior to walking onset 2. More widespread activation in stroke group compared to healthy young adults (from Sburlea et al. [29]). Though no statistical comparisons made

All results are reported in comparison to baseline activation (typically standing prior to walking)

*ROI*  region of interest, *PFC*  prefrontal cortex, *SMA* supplementary motor area, *SMC*  sensorimotor cortex, *M1*  primary motor cortex, *fNIRS*  functional near-infrared spectroscopy, *EEG*  electroencephalography

*Sburlea et al. [46] was included with their 2015 publication as they used the same data set in both studies

None of these studies compared brain activation to gait performance.

**Fig. 3:**
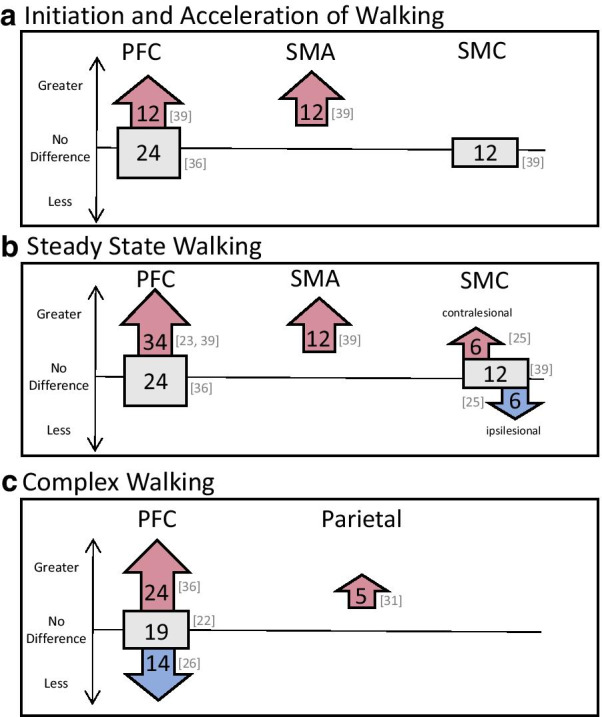
Differences in regional activation patterns in comparison to age-matched healthy individuals. Arrows pointing up indicate greater activation, arrows pointing down indicate less activation, and squares indicate no difference between stroke groups and age-matched healthy adults. Numbers within the shapes represent total number of participants within the studies, with the specific studies cited to the right of the shapes. Panel **a**, **b**, and **c** represent studies looking at the initiation/acceleration phase of walking, steady-state phase of walking, and complex walking tasks, respectively

When compared to young adults, PFC increases were greater in the stroke group [36]; in a follow-up study, Sburlea et al. [46] reanalyzed their dataset and showed that brain activation increases were similar but more widespread (i.e., larger volume of activated areas) in the stroke group. PFC activations compared to older adults were less clear. Hawkins et al. [36] showed similar PFC increases between their chronic stroke group and older adults while Mihara et al. [39] showed greater activation over right PFC in their subacute stroke group with ataxia compared to older adults. Mihara et al. [39] also showed greater activation over SMA for their ataxic group but no difference in SMC activation compared to older adults (Fig. 3a).

#### Brain activation during steady-state walking

Fifteen studies investigated brain activity during steady-state walking [23–25, 27, 28, 30, 33–37, 39, 41–43] (Table 2). All results will be described as activations during walking in comparison to standing immediately prior to walking, unless otherwise stated. Overall, stroke participants showed bilateral activations in PFC, PMC, SMA, SMC, superior parietal and occipital lobe and greater activations were found in the contralesional hemisphere for SMC and parietal areas (Fig. 2b). A variety of steady-state walking tasks were compared. These included:

- single-session unassisted overground or treadmill walking [23, 25, 27, 28, 33, 34, 36, 37, 39],
- single-session assisted walking with sensory feedback [28], walking with body-weight support [25], and walking with robotic [23, 30, 35] or therapist [24] assistance,
- multi-session gait interventions: controlled [41] and uncontrolled [42, 43].

**Table 2:** Studies investigating steady-state walking listed by increasing walking speed

Authors	Device [ROI]; method of placing channels	Walking time analyzed, task and speed: m/s (SD)	Results
(Miyai et al., 2003) [43]	fNIRS [PFC, PMC, pre-SMA, SMA, SMC]; relative to Cz. ROI based on MRI of 2 subjects	10–30 s post-start, Treadmill with body weight support: 0.06	1. Baseline: decreased ipsilesional SMC activation compared to contralesional SMC 2. Two-months post inpatient rehab: increased SMC symmetry, greater ipsilesional PMC activity and increased cadence, gait symmetry, Fugl-Meyer LE score 3. Greater pre-SMA and PFC activation in patients with large cortical lesions for both time points. No activation over lesioned cortex 4. Increased SMC symmetry (not PMC, SMA) correlated to increased gait symmetry
(Miyai et al., 2002) [24]	fNIRS [PFC, PMC, pre-SMA, SMA, SMC]; same as Miyai et al. 2003	10–30 s post-start, Treadmill with manual assistance at the leg or facilitation at hip: 0.06	1. Increased bilateral PFC, PMC, pre-SMA, and more prominent contralesional SMC (compared to ipsilesional SMC) activation 2. Greater bilateral pre-SMA and PFC, ipsilesional PMC activation, increased cadence and gait symmetry with facilitation at the hip compared to manual assistance of leg 3. No activation over lesioned cortex
(Contreras-Vidal et al., 2018) [42]	EEG [Whole head]; 10/20 system	10–360 s post-start, Overground, comfortable pace with exoskeleton: (0.14–0.31)*	1. Increased activation over frontal and central regions 2. Consistent pre and post training activation over occipital regions 3. Gait speed doubled after 12 sessions of exoskeleton gait training
(Sangani et al., 2015) [28]	fNIRS [PFC, PMC, SMA, SMC]; relative to Cz	15–25 s post-start, Treadmill, comfortable pace with and without light finger touch: 0.21	1. Increased activation along frontal and sensorimotor cortices with greater activation along contralesional hemisphere 2. Greater overall activation symmetry, localized activation to SMC, and increased gait speed with light finger touch
(Miyai et al., 2006) [25]	fNIRS [PMC, SMA, SMC]; same as Miyai et al. 2003	10–20 s post-start, Treadmill with and without body weight support (BWS): Stroke: 0.30 Healthy: 0.30	1. Overall increased bilateral PMC and SMA, contralesional SMC activity 2. Increased SMC and gait symmetry with BWS 3. Increased bilateral PMC, SMA, SMC for healthy participants and a general increase in activation with BWS 4. For stroke only: increased SMC symmetry correlated to increased gait symmetry and increased SMC (not PMC, SMA) activity correlated to increased cadence
(Mihara et al., 2007) [39]	fNIRS [PFC, SMA, SMC]; same as Miyai et al.2003	24-30 s post-treadmill start, Treadmill: Stroke: Fast, comfortable pace, 0.33 (0.22) Healthy: Comfortable pace, 0.97 (SD not provided)	1. Increased PFC, SMA, and SMC activity 2. Compared to the acceleration phase, PFC and SMA activation decreased in healthy and increased in the stroke group 3. SMC activation remained similar throughout walking for both groups
(Garcia-Cossio et al., 2015) [35]	EEG [Whole head]; 10/20 system	During “constant and stable speed”, Treadmill, comfortable speed with exoskeleton: Stroke: max 0.42 Healthy: 0.42	1. Increased activation at fronto-central and contralesional centro-parietal regions 2. No gait-cycle related modulations in brain activity compared to healthy adults who showed low gamma fluctuations with gait-cycle 3. No correlations between brain activity and walking performance
(Hawkins et al., 2018) [36]	fNIRS [PFC]; high and lateral on the forehead	37–67 s after start command, Overground, preferred speed: Stroke: 0.51 (0.27) Older adults: 1.07 (0.16) Young adults: 1.28 (0.18)	1. Increased PFC activity 2. Greater increase in PFC compared to young adults, no different to older adults
(Hermand et al., 2019) [37]	fNIRS [PFC]; 10/20 system	10–20 s post-start, Overground, comfortable pace: 0.52 (0.23)	1. Increased PFC activity 2. No differences between hemispheres 3. No significant correlations between brain activation and gait performance
(Chatterjee et al., 2019) [33]	fNIRS [PFC]; high and lateral on forehead	7–37 s post-start, Overground, self-selected: 0.6 (0.2)	1. Increase PFC activation during walking 2. Low balance confidence subgroup showed greater PFC, slower walking speed, and shorter stride length (not step width) compared to high balance confidence subgroup 3. No differences between hemispheres
(Chen et al., 2019) [41]	EEG [Whole head]; 10/20 system	0–300 s post-start, Treadmill, Time point 1: 0.58 (0.23) Time point 2: 0.71 (0.31)	1. Increase connectivity between contralesional frontocentral, middle central and ipsilesional centroparietal, greater increase in gait speed and temporal symmetry post turning treadmill 2. Increased connectivity correlated to increased gait symmetry but not gait speed 3. No differences in connectivity after regular treadmill training
(Choi et al., 2016) [34]	EEG [Whole head]; 10/20 system	Entire walking task (10–20 s long), Overground, self-paced: speed not reported	1. Increased activation in contralesional centro-parietal area
(Lee et al., 2018) [30]	fNIRS [PFC, PMC, SMA, SMC]; not reported	5–30 s post-start, Treadmill, self-selected with exoskeleton: speed not reported	1. Decreased SMA, PMC, SMC activity with versus without robotic assistance compared to no assistance
(Caliandro et al., 2020) [23]	fNIRS [PFC]; 10/20 system	5 s to end of walking, Overground, with and without exoskeleton: speed not reported	1. Both groups: greater PFC activation for walking with versus without exoskeleton 2. Greater PFC activation for the stroke compared to healthy group 3. No hemispheric difference
(Saitou et al., 2000) [27]	fNIRS [Ipsilesional forehead]; “forehead of impaired side”	0–300 s post-start, Overground: speed not reported	1. Increased activation for 15 out of 22 participants (cerebral blood volume and cerebral oxygenation volume)

All results are reported in comparison to baseline (unless otherwise stated)

*ROI*  region of interest, *PFC* prefrontal cortex, *PMC*  premotor cortex, *SMA*  supplementary motor area, *SMC*  sensorimotor cortex, *M1*  primary motor cortex, *fNIRS*  functional near-infrared spectroscopy, *EEG*  electroencephalography

*Interpreted from graph

*Unassisted walking:* Increased bilateral activations were observed in PFC (6 of 8 studies: [23, 28, 33, 36, 37, 39]), PMC (2 studies: [25, 28]), and SMA (3 studies: [25, 28, 39]) for the stroke groups. These studies, with the exception of Sangani et al. [28], showed no differences between hemispheres. Sangani et al. [28] found greater overall contralesional activation; however, this was a single-subject proof-of-concept study and specific details of precise activations were not reported. Saitou et al. [27] only measured from ipsilesional PFC and showed increased activation during walking compared to standing in 15 of the 22 patients tested. Increased PFC activations were also related to greater impairment (e.g., lower Fugl-Meyer scores) [33, 36] and lower balance confidence [33].

Increases in brain activity during walking were also found in SMC (4 studies: [25, 28, 34, 39]) and parietal regions (1 study: [34]). In both these areas, more studies found greater activation in the contralesional hemisphere with either little or no activation in the ipsilesional hemisphere [25, 28, 34].

Compared to young healthy adults, PFC activation in stroke was greater [36]. However, compared to older adults, Hawkins et al. [36] showed similar activations in both groups. Conversely, greater PFC activation was reported for the stroke group in two studies [23, 39]. Mihara et al. [39] also showed a sustained elevation in activation over SMA regions in their ataxic stroke group and decreased PFC and SMA activation in their healthy adult group during steady-state compared to acceleration. No between group differences were observed for SMC [39] (Fig. 3b).

*Assisted walking:* Increased PFC was observed during overground exoskeleton walking compared to overground unassisted walking [23]. No difference in PMC and SMA activations were found between body weight supported walking and unassisted treadmill walking [25]. In contrast, Lee et al. [30] found an overall decrease in PMC, SMA, and SMC activation with robotic assistance compared to unassisted overground walking. Compared to unassisted walking, 2 of the 3 studies showed increased brain activation symmetry over the SMC during body weight supported walking [25] and with light finger touch on a stable surface [28] compared to unassisted walking. Increased symmetry in SMC activation was related to increased gait symmetry [25, 28] and increased SMC activity also related to increased walking cadence [25]. No correlations were found with PMC or SMA activations with gait performance.

Robotic walking, compared to upright stationary body-weight suspension, resulted in increased bilateral activation over SMC and contralesional centro-parietal regions [35]. Manual assistance of the paretic leg during gait, compared to standing, resulted in increased bilateral activation of PFC, PMC, SMA, and SMC, with greater activation in ipsilesional (compared to contralesional) PMC and contralesional (compared to ipsilesional) SMC [24]. Miyai et al. [24] also compared manual assistance of the leg to facilitation at the hip and found greater overall activations and greater symmetry of SMC activity with facilitation. Facilitation also resulted in increased walking cadence and symmetry [24]. No correlations were found between brain activations during robotic walking and walking performance [35].

In healthy individuals, young adults showed differential activations based on gait-phase whereas no phasic activations were observed after stroke [35]. In older adults, similar regions were activated compared with the stroke groups, though no asymmetries were observed [25, 28] (Fig. 3b).

*Multi-session gait interventions*: Interventions took place with chronic stroke groups 3 times a week for 4 weeks [41, 42] and in subacute individuals during inpatient rehabilitation for 2 months [43]. Prior to these interventions, individuals who were not walking independently showed minimal activation over ipsilesional SMC during body weight supported treadmill walking [43] and individuals who were able to walk independently showed broad activations over SMA and occipital lobe when first using robotic assistance [42]. After turning treadmill training, Chen et al. [41] showed increased connectivity with the middle central, contralesional frontocentral, and ipsilesional centroparietal regions, whereas no changes in connectivity were observed in the group that received regular treadmill training. After overground robotic gait training, Contreras-Vidal et al. [42] showed greater localization of brain activation to SMA and occipital lobe. With treadmill-based gait rehabilitation, increased activation in ipsilesional SMC and PMC were observed [43]. Miyai et al. [43] also found increased PFC and SMA activation that persisted throughout rehabilitation for participants with large cortical strokes and severe hemiparesis. Increased SMC brain activations and connectivity were related to increased gait symmetry [41, 43] but not gait speed [41] though Contreras-Vidal et al. [42] showed a doubling in gait speed after their intervention. No correlations were found with PMC or SMA [43]. No healthy adults were included in these intervention studies.

#### Brain activation during complex walking

Nine studies investigated brain activation during complex walking (Table 3). Investigation of PFC activation was the most common region of interest amongst the studies (7 studies) whereas only one or two studies investigated PMC, SMA, SMC, parietal and occipital regions. The majority of these studies used an additional cognitive or motor task (i.e. dual-task walking) to increase the complexity of walking (6 studies: [22, 26, 33, 36–38]). Other studies investigated externally cued walking (i.e., real-time direction of where and how to walk) using virtual reality [40], augmented reality [32] or objects on the ground [31]. All studies, with the exception of Calabro et al. [40], compared their complex walking task to simple, overground walking. Calabro et al. [40] compared their complex walking paradigm to linear exoskeleton walking on a treadmill. The following detailed results are described as comparisons to each study’s simple walking task.

**Table 3:** studies investigating complex walking listed by increasing walking speed

Authors	Device [ROI]; method of placing channels	Walking task and speed: m/s (SD)	Method of adding complexity to walking + comparator condition	Results
(Mitchell et al., 2018) [31]	[18F]-FDG-PET [Whole head]; spatial resolution 4.8–5.4 mm	Overground, comfortable pace: *Stroke: complex/comparator 0.45(0.26)/0.76(0.42) *Healthy: complex/comparator 0.81(0.14)/1.34(0.04)	*Complex:* Overground obstacle course *Comparator:* Overground walking	1. More asymmetric activation in superior parietal areas compared to healthy 2. More impaired showed greater activation in ipsilesional PFC and contralesional superior parietal 3. Less impaired showed greater activation in contralesional PFC and ipsilesional superior parietal 4. No subcortical differences between stroke and healthy*
(Al-Yahya et al., 2016) [22]	fNIRS [PFC]; 10/20 system	Treadmill, comfortable pace: Stroke: 0.48 (0.338) Healthy: 1.01 (0.025)	*Complex:* Cognitive dual-task (serial 7 subtractions) *Comparator:* treadmill walking	1. Greater PFC activation 2. No difference compared to older adults 3. No difference between hemispheres
(Hawkins et al., 2018) [36]	fNIRS [PFC]; high and lateral on the forehead	Overground, preferred speed: Stroke: 0.51 (0.27) Older: 1.07 (0.16) Younger: 1.28 (0.18)	*Complex:* Motor and cognitive dual-task (obstacle stepping and verbal fluency) *Comparator:* Overground walking	1. Greater increase in PFC activity with complex walking compared to young and older adults 2. Greater PFC activation in those with greater impairment (Fugl-Meyer LE), despite having similar gait speeds
(Hermand et al., 2019) [37]	fNIRS [PFC]; 10/20 system	Overground, comfortable pace: 0.52 (0.23)	*Complex:* Cognitive dual-task (2 difficulties: verbal 1-back and 2-back) *Comparator:* Overground walking	1. No change in PFC activation 2. Decreases in walking speed and gait variability 3. No difference in gait performance between difficulty of complex walking 4. Decrease cognitive performance with harder compared to easier complex walking 5. No significant correlations
(Chatterjee et al., 2019) [33]	fNIRS [PFC]; high and lateral on forehead	Overground, self-selected: 0.6 (0.2)	*Complex:* Cognitive dual-task (serial 7 subtraction) *Comparator:* Overground walking	1. Greater PFC activation, slower gait speed, shorter stride length, wider step width 2. Higher cognitive status and lower extremity impairment predicted greater PFC activation during complex walking 3. Low cognitive status subgroup: no change in PFC activation, decreased gait speed, shorter stride length 4. Greater PFC cost of complex walking correlated to greater decreased in walking speed and stride length
(Liu et al., 2018) [38]	fNIRS [PFC, PMC, SMA]; no detail on head cap placement	Overground, self-selected: 0.74–0.60 (0.16–0.18)	*Complex:* Motor and cognitive dual-task (water bottle on tray, serial 3 subtraction) *Comparator:* Overground walking	1. Manual dual-task: greater increase in bilateral PMC and contralesional SMA 2. Cognitive dual-task: greater increase in ipsilesional PFC, bilateral PMC, and contralesional SMA 3. Greater PMC and SMA (but not PFC) activity correlated to lower cadence, greater stride time, and slower walking speed
(Mori et al., 2018) [26]	fNIRS [PFC]; landmarked to Fp1 and Fp2	Overground, comfortable pace: Stroke: 0.94 (0.23) Healthy: 1.24 (0.18)	*Complex:* Cognitive dual-task (serial 3 subtraction) *Comparator:* Overground walking	1. Less PFC activation and greater gait and cognitive costs (i.e. worse performance) in the stroke compared to healthy 2. Stroke: Greater right PFC correlated to better dual-task gait performance 3. Healthy: Greater left PFC correlated to better cognitive performance
(Calabro et al., 2017) [40]	EEG [whole head]; 10/20 system and LORETA source localization	Treadmill: 1.8	*Complex:* 8 weeks exoskeleton, non-linear force + virtual environment *Comparator:* 8 weeks exoskeleton linear walking, on-screen smile feedback	1. Greater ipsilesional fronto-central, M1, S1, and visual cortex activity, gait cycle dependent changes, gait/balance performance, and hip/knee force 2. Both conditions: decreased depression, no spasticity change 3. Increased frontal and central brain activations correlated to increased gait and balance performance and hip force 4. No correlations between brain activity and age, gender, stroke duration, or number of comorbidities
(Chang et al., 2019) [32]	EEG [Cz (SMC)]; not reported	Overground, pace not reported	*Complex:* Augmented reality walking with music + stepping targets *Comparator:* Overground walking, no music or targets	1. Greater SMC activation 2. Greater hip and knee flexion

All results are reports as changes in complex walking from the comparator condition

*ROI*  region of interest, *PFC*  prefrontal cortex, *PMC*  premotor cortex, *SMA*  supplementary motor area, *SMC*  sensorimotor cortex, *M1*  primary motor cortex, *fNIRS*  functional near-infrared spectroscopy, *EEG*  electroencephalography, *[18F]-FDG-PET*  ^18^F-labeled fluoro-2-deoxyglucose positron emission tomography

*Details obtained through personal communication with authors

With *dual-task walking*, five groups solely investigated PFC activity [22, 26, 33, 36, 37] and one group investigated PFC, PMC, and SMA [38]. Four of these six studies showed increased PFC activity with dual-task walking [22, 33, 36, 38]. Two studies showed no change in PFC activation with dual-task, though other characteristics were noted: Hermand et al. [37] showed significant decreases in walking speed and increased gait variability with dual-task walking, and although Mori et al. [26] also showed no group change in PFC activity, they found that more PFC activity correlated to less change in gait acceleration magnitude (i.e., less walking-related detriments). On the contrary, Chatterjee et al. [33] showed that greater PFC change was related to greater decreases in walking speed and stride length (i.e., greater walking-related detriments). Hawkins et al. [36] completed a subgroup analysis and found that those with greater impairment (i.e., lower Fugl-Meyer scores) showed greater PFC activation compared to individuals with less impairment; there were no differences in gait speed for these subgroups. Liu et al. [38] also showed increases in bilateral PMC and contralesional SMA with dual-task walking (Fig. 2c). These increased activations were correlated to decreased walking speed and cadence, and increased stride time and asymmetry (i.e., worse walking performance).

In comparison to young adults, a larger increase in PFC activity was observed for the stroke group [36]. In contrast, comparisons with older adults were variable and showed greater [36], less [26] and similar [22] PFC activations (Fig. 3c).

*Externally cued walking* generally resulted in increased activations over PFC [31], PMC [40], SMA [40], SMC [32, 40], parietal areas [31, 40], and occipital areas [40] when compared to non-cued walking. Specifically, using special glasses to virtually cue stepping with music resulted in significant increases in activation over SMC compared to overground, non-cued walking [32]. An 8 week, 5 sessions per week exoskeleton intervention using complex, obstacle navigation in virtual reality led to greater ipsilesional PMC, SMA, and SMC activations, bilateral parieto-occipital activations, and distinct activation patterns related to the gait-phase compared to linear exoskeleton gait training with no virtual environment [40]. Different stroke severities also resulted in different asymmetric activations during complex walking: greater activations were observed over ipsilesional PFC and contralesional parietal areas for more impaired individuals; less impaired individuals showed more activation over contralesional PFC and ipsilesional parietal areas [31]. Increased ipsilesional SMA activation correlated to increased gait and balance performance [40]. No significant correlations were found between brain activations and age, sex, stroke duration or number of comorbidities [40]. Compared to healthy age-matched adults, more asymmetrical activation was observed over superior parietal regions in the stroke group [31].

## Discussion

This is the first review to consider patterns of spatial and temporal brain activation during different components of real-time walking in individuals with stroke. Overall, compared to standing, all components of walking generally showed increased activation across all areas of the brain that were measured: PFC, PMC, SMA, SMC, parietal, and occipital regions. Distinct differences in symmetry of activation were observed between walking components which depended on brain region and gait performance. Comparisons to healthy individuals were variable and depended on the age of the comparator group, the region of interest, and walking category. Possible explanations for asymmetries and between group comparisons are discussed below.

### Asymmetric activations

Previous studies and reviews have typically shown symmetrical brain activations in healthy young and older adults for gait preparation, steady-state, and complex gait [10, 48]. Our systematic review suggests that activation symmetry may be a biomarker of walking recovery after stroke with activation asymmetry correlating to asymmetrical gait biomechanics and poorer gait performance. Further, our results suggest that rehabilitation therapies which allow more symmetrical motor performance (e.g., body weight support, sensory feedback using light touch, therapist facilitation or via multiple training sessions) may improve ipsilesional SMC activation, and consequently SMC symmetry.

In the upper extremity, greater contralesional activation has been attributed to activation from the uncrossed corticospinal tract [49], compensatory networks to facilitate ipsilesional movements [50] or an increase in relative interhemispheric inhibition from the contralesional to ipsilesional hemisphere [51]. Functional recovery of the paretic limb has then been associated with either increased activation in the ipsilesional hemisphere or increased activation in motor related areas of the ipsi- and contralesional hemisphere [52, 53]. The current literature in the lower extremity point to some differences [54] and similarities [55] in recovery mechanisms compared to the upper extremity. Although, distinct mechanisms of lower limb recovery remain unclear, we can speculate that similar models could be applied to the lower extremity.

Within this current review, the observed asymmetries towards the contralesional hemisphere—particularly in SMC—appeared to decrease with gait interventions, however, the asymmetries appeared to persist for individuals with severe walking difficulties [35], large cortical strokes [43] and slower walking speed (i.e., slower than 0.5 m/s) [24, 25, 28, 43]. It is possible that if the structures involved in motor output (e.g., SMC and corticospinal tract) are severely damaged, there may be limited recovery potential in that region and greater activation in association areas are needed to compensate [56].

Activations within complex walking studies were highly variable with half of the studies showing asymmetrical activations. The laterality of these asymmetries tended to favour the ipsilesional hemisphere, though increased contralesional activations in specific subgroups and tasks were also observed [31, 38]. The differences between each of these tasks make it difficult to generalize the findings and no obvious differences in study design, stroke population, or region of interest are present between studies that do and do not show brain activation asymmetries. Thus, it is currently unclear under which circumstances complex walking results in asymmetries and how these asymmetries should be interpreted.

Finally, it is important to note that no asymmetries were reported for the studies looking at the initiation or acceleration phases of walking. While motor planning in the lower extremity is not well studied post-stroke, the limited work available supports the findings within this review. Peters et al. [57] showed similar pre-movement EEG potentials when the paretic or non-paretic leg was used to step onto a box. This lack of difference between paretic and non-paretic limbs and lack of brain asymmetry may suggest that the planning or initial brain activation associated with walking may not be impaired post-stroke.

### Brain activations compared to healthy adults

This review found a consistent increase in brain activation in steady-state walking compared to younger adults but mixed results in comparison to older adults, which may suggest that the increased activation is a function of age and not necessarily an effect of stroke. This is consistent with previous reviews and studies showing greater brain activity in healthy older adults [58], and individuals with Parkinson’s Disease [12, 59] when compared to younger adults. However, more studies are needed to explore this hypothesis. Studies on complex walking did not find consistent results, and this may be due to differing tasks, as well as variability in stroke chronicity. With less than half the studies making direct comparisons between the stroke group and a group of healthy adults, it is difficult to make any conclusions about how brain activation may differ post-stroke.

### Limitations within the literature

There are numerous common limitations within the studies in this review. First, the majority of studies involved less than 20 participants. This poses a large problem as the between-subject variability (though not commonly reported) is likely very high within these brain activation methods [60]. This large between subject variability likely contributes to the discrepancies in results. Additionally, less than half the studies made comparisons with a healthy age-matched group. Direct comparisons with healthy adults are important to fully understand if brain activation differs after stroke, or if it is a function of the aging process. Previous works suggest brain activations differ with aging and depending on the specific task [12]. Without a direct comparison with a healthy older adult group, no clear comparison to “normal” brain activations can be made. Furthermore, it is important to note that while most participants were tasked with similar walking goals (i.e., comfortable walking), the actual speed of walking between studies and between groups within the same study often differed. Previous studies have shown some scaling of brain activation with gait speed in neurological populations [61] though no specific investigations have been made in the stroke population. Although not discussed in detail within this review, the included studies used many different time windows for data analysis. Several studies used the timeframe of the entire walking task to assess brain activation, while others separated the acceleration or early phase of walking from the steady-state phase. Evidence from one study that separated walking phases showed differences in activation for each phase [39]; so, it is possible that separating brain activation by phase of walking may result in different findings.

Within the fNIRS studies, the majority of the newer studies solely investigated the PFC region [22, 23, 27, 33, 36, 37]. Investigation of brain activation beyond the PFC, particularly the parietal cortex, is important as several EEG and [18F]-FDG-PET studies suggest increased activation over the parietal lobes during walking. Finally, the method of placing channels to assess regions of interest is overall poor or severely under reported. Individual brain morphology, especially with aging and after stroke, is highly variable [60] and more precise methods are required to accurately measure ROIs. Most studies used rough estimates based on a few skull landmarks to then align a headcap or band embedded with channels. This, on its own, is problematic—especially for studies with multiple ROIs—as it does not ensure that similar regions are being recorded between participants or within participants across several sessions. Technologies that allow for 3D digitization of channels and subsequent co-registration to atlas brains or individual structural anatomy can improve the accuracy and consistency in channel placements. None of the studies included within this review digitized their channels, and only four studies defined their channels based on structural anatomy from representative subjects [24, 25, 39, 43].

### Limitations of this review

Due to the infancy of this field, we included data from all types of studies including book chapters and conference abstracts. While the inclusion of non-peer reviewed studies may affect the quality of the data, we believe it was important to include all available data due to the limited number of published studies within this field. Along with using the NIH Study Quality Assessments (Appendix Table 6), information displayed as total number of subjects (Fig. 2) may further inform the reader on the possible strength of a finding. In addition, the inclusion of multiple brain recording modalities makes it difficult to consolidate information and thus quantitative analysis of the findings was not possible. Due to this broad inclusion, consolidation of brain activation across sites arising from different modalities should be taken with caution. However, the inclusion of these modalities has shown the need for more exploration, especially with fNIRS, in more posterior cortical regions.

## Conclusion

By separating brain activation results based on walking categories, our findings showed distinct activation differences and apparent limitations within the current literature. Symmetrical increases in motor planning and execution areas (i.e., PFC, SMA, and SMC) were activated for initiation/acceleration. Half the studies showed greater contralesional activation in motor execution and sensory integration areas (i.e., SMC and parietal regions) during steady-state, which was more apparent at slower walking speeds and related to gait performance. A less distinct tendency toward ipsilesional activations with complex walking was also observed. Individuals post-stroke employed greater brain activation compared to young adults, while comparisons to older adults were less clear. With these findings we make the following recommendations for future studies:

1. Larger sample sizes (n > 20) of more homogeneous stroke participants (i.e., severity, lesion side) are needed to account for the large inter-subject variability in brain imaging data
2. Direct, controlled comparisons with healthy age-matched adults should be made
3. Time frame of data analyzed should take into account the different phases of gait (i.e., do not group together the acceleration and steady-state phases)
4. Stroke location should be accounted for or detailed reports are needed in how the data is handled when measuring over lesion locations.

## Data Availability

All information found within this review have been taken from previously published data. Further detail on studies that have not been published in a peer-review paper have been obtain from personal communication with the authors.

## Appendix

See Tables 4, 5, 6.

**Table 4:** Detailed search strategy from 6 databases

		Medline (Ovid)	Embase (Ovid)	Pubmed	Web of Science	CINAHL (EBSCOHOST)	PsycInfo (EBSCOHOST)
P: Adult stroke	MESH	Cerebrovascular disorders Brain ischemia (exp) Stroke (exp)	Exp cerebrovascular accident/ Exp brain ischemia/	Stroke *Stroke rehabilitation* *Lacunar stroke* Cerebrovascular disorders Cerebrovascular trauma	No mesh	Cerebrovascular disorders + Intracranial thrombosis + Intracranial embolism + Intracranial embolism and thrombosis + Stroke + Intracranial hemorrhage +	DE "Cerebrovascular Accidents" DE "Cerebrovascular Disorders" DE "Cerebral Ischemia" DE "Cerebral Hemorrhage"
	Keywords	((cerebrovascular or cerebral or brain) adj3 (ischemia or accident* or stroke* or disease* or disorder*)) or CVA* or stroke* or (((cerebrovascular or cerebral or brain) adj3 (ischemia)) or ((cerebrovascular) adj3 (accident* or disease* or disorder*)) or CVA* or stroke*)		((cerebrovascular) AND (ischemia OR infarct* OR accident* OR stroke* OR disease* OR disorder*)) OR CVA* OR stroke* OR (cerebral AND infarct*)		((cerebrovascular or cerebral or brain) n3 (ischemia or accident* or stroke* or disease* or disorder*)) or CVA* or stroke*	
I: Walking	MESH	Locomotion Walking Gait	Locomotion Walking Gait	Locomotion Walking Gait	No mesh	Locomotion Walking Gait	DE “Locomotion” DE “Walking” DE “Gait”
	Keywords	(Ambulat* OR gait OR walk* OR locomot*)					
O: brain activation	MESH	Neuroimaging Functional neuroimaging Brain mapping Spectroscopy, near-infrared Electroencephalography Electrocorticography Tomography, Positron-emission Tomography, emission-computed, single-photon	Neuroimaging Functional neuroimaging Brain mapping near-infrared Spectroscopy Functional near-infrared Spectroscopy Electroencephalography Electrocorticography Positron emission tomography Positron emission tomography computed tomography Single-photon emission-computed tomography	Neuroimaging Functional neuroimaging Brain mapping Electroencephalography Electrocorticography Positron emission tomography Positron emission tomography-computed tomography Single-photon emission-computed tomography	No mesh	Spectroscopy, near-infrared Electroencephalography + Tomography, Emission-Computed + Tomography, Emission-Computed, Single-Photon +	DE "Neuroimaging" DE "Electroencephalography" " DE "Positron Emission Tomography" DE "Single Photon Emission Computed Tomography"
	Keywords	((Functional or brain or cort* or cerebr* or motor or sensory or sensorimotor or prefrontal or frontal or neural) adj3 (neuroimag* or activ* or tomography or correlate* or map*)) or neuroimag* or near-infrared spectroscopy or FNIRS or electroencephalograph* or EEG or electrocorticograph* or ECOG or positron-emission tomography or PET or single-photon emission-computed tomography or SPECT		(((Functional OR brain OR cort* OR cerebr* OR motor OR sensory OR sensorimotor OR prefrontal OR frontal OR neural) AND (neuroimag* OR activ* OR tomography OR correlate* OR map*)) OR neuroimag* OR near-infrared spectroscopy OR FNIRS OR electroencephalograph* OR EEG OR electrocorticograph* OR ECOG OR positron-emission tomography OR PET OR single-photon emission-computed tomography OR SPECT)		((Functional or brain or cort* or cerebr* or motor or sensory or sensorimotor or prefrontal or frontal or neural) n3 (neuroimag* or activ* or tomography or correlate* or map*)) or neuroimag* or near-infrared spectroscopy or FNIRS or electroencephalograph* or EEG or electrocorticograph* or ECOG or positron-emission tomography or PET or single-photon emission-computed tomography or SPECT	
Limits		All adults (19 plus years), English					

**Table 5:** Study participant characteristics

Study	Number of participants	Age	Sex [F/M]	Time post-stroke [months (SD)]	Type of stroke	Lesion location	Walking status
(Al-Yahya et al., 2016) [22]	19 Stroke; 20 Healthy	59.61 (15.03); 54.35 (9.38)	2/17; 8/12	Chronic: 26.5 (27.46)	NR	11 Right; 8 Left	Independent
(Calabro et al., 2017) [40]	24 Stroke (12/group)	60 (4); 63 (6)	5/7; 5/7	Chronic: 7.92 (1.95)	Ischemic	13 Right; 11 Left 4 F&P; 4 P&O; 4 T&P; 6 P; 6 F	FAC 0–4
(Caliandro et al., 2020) [23]	22 Stroke 15 Healthy	47–74; 43–69	9/13; 6/9	Chronic: 55.6 (36.6)	Ischemic	12 Right; 10 Left 3 F; 10 F, T, P & IC; 4 IC; 2 F & IC; 2 T & IC; 1 F & P	Independent
(Chang et al., 2019) [32]	2 Stroke	NR	NR	NR	NR	NR	Independent
(Chatterjee et al., 2019) [33]	33 Stroke	59.6 (9.7)	11/22	Chronic: 19.2 (10.4)	NR	17 Right; 16 Left 4 ACA; 10 MCA; 14 BG & IC; 5 Pons	Moderate-severe walking deficits (walking speed < 0.8 m/s)
(Chen et al., 2019) [41]	18 Stroke (9/group)	50.33 (10.95); 54.67 (8.32)	1/8; 0/9	Chronic: 35.63 (27.42)	9 Hemorrhagic 9 Ischemic	6 Right; 12 Left	Independent
(Choi et al., 2016) [34]	3 Stroke	62 (9)	1/2	Chronic: 10.3 (8.08)	1 Hemorrhagic 2 Ischemic	2 Right; 1 Left	Independent
(Contreras-Vidal et al., 2018) [42]	5 Stroke	50.6 (11.46)	0/5	Chronic: 65 (65.68)	2 Hemorrhagic 2 Ischemic 1 Mixed	4 Right; 1 Left 1 F,T&P; 1 Th; 1 F & P; 2 unclassified	Independent
(García-Cossio et al., 2015) [35]	3 Stroke; 10 Healthy young adults	46.7 (16.9); 32.3 (10.8)	0/3	Sub-acute: 2.33(0.56)	3 Ischemic	3 Right	“severe difficulties to stand and walk”
(Hawkins et al., 2018) [36]	24 Stroke; 15 Healthy older adults; 9 Healthy young adults	58 (9.3); 77.2 (5.6); 22.4 (3.21)	8/16; 8/7; 5/4	Chronic: 18.3(9.3)	NR	NR	“Moderate-severe gait impairment” (based on walking speed < 0.8 m/s)
(Hermand et al., 2019) [37]	11 Stroke	71.4 (10.1)	NR	Sub-acute: 1.52 (1.15)	2 Hemorrhagic 9 Ischemic	5 Right; 6 Left MCA	Independent
(Lee et al., 2018) [30]	20 Stroke	61.74 (6.93)	7/13	Chronic: 36.67 (26.61)	14 Hemorrhagic 6 Ischemic	12 Right; 8 Left 1 cortical; 10 subcortical; 9 mixed	NR
(Liu et al., 2018) [38]	23	51.5 (10.7)	2/21	Chronic: 41.5 (41.4)	11 Hemorrhagic 12 Ischemic	11 Right; 12 Left	Independent
(Mihara et al., 2007) [39]	12 Stroke; 11 Healthy	52.7 (16.9); 42.6 (11.6)	1/11; NR	Sub-acute: 2.94 (1.49)	6 Hemorrhagic 6 Ischemic	6 Left cerebellum; 1 Right cerebellum; 1 Bilateral cerebellum; 2 Left medulla; 2 Right pontine tegmentum	Independent
(Mitchell et al., 2018) [31]	5 Stroke; 7 Healthy	NR; Age-matched	NR	Chronic: NR	NR	Side NR IC & BG	Independent
(Miyai et al., 2006) [25]	6 Stroke; 5 Healthy	57 (6); 53 (11)	1/5	Sub-acute: 2.5 (0.9)	2 Hemorrhagic 4 Ischemic	2 Right; 4 Left 2 IC & Th; 3 CR; 1 CR & Pt	Independent or with supervision
(Miyai et al., 2003) [43]	8 Stroke	57 (12)	3/5	Sub-acute: Time 1 = 2.7 (1) Time 2 = 5.3 (1)	4 Hemorrhagic 4 Ischemic	4 Right; 4 Left 5 subcortical only; 1 cortical only; 2 mixed	Dependent
(Miyai et al., 2002) [24]	6 Stroke	57 (13)	2/4	Sub-acute: 2.7 (1.03)	4 Hemorrhagic 2 Ischemic	2 Right; 4 Left 3 P & CR; 2 Pt, CR, IC; 1 F, P, T, Pt, CR & IC	Maximum assist
(Mori et al., 2018) [26]	14 Stroke; 14 Healthy	61.1 (9.3); 66.3 (13.3)	2/12	Chronic: NR	9 Hemorrhagic 5 Ischemic	8 Right; 6 Left Excluded PFC lesions 2 IC; 3 Th; 8 Pt; 1 CR	Independent
(Saitou et al., 200)0 [27]	44 Stroke *only 22 completed walking task*	66 (9.3)	13/31	Chronic: 34.27 (6.57)	11 Hemorrhagic 33 Ischemic	25 Right; 19 Left 6 Pt; 4 Th; 1 T P; 27 penetrating branches; 6 MCA	Independent
(Sangani et al., 2015) [28]	1 Stroke	71	1	Chronic: 120	NR	Left MCA	Independent
(Sburlea et al., 2015) [29]	9 Stroke; ***10 Healthy young adults	59.7 (11.3); 26.4 (4.8)	3/6; 4/6	Chronic: 75.76 (67.61)	3 Hemorrhagic 6 Ischemic	NR	Independent (not explicitly reported)

*NR* not reported, *F* frontal, *P* parietal, *O* occipital, *T* temporal, *ACA* anterior cerebral artery, *MCA* middle cerebral artery, *BG* basal ganglia, *IC* internal capsule, *Th* thalamus, *CR* corona radiata, *Pt* Putamen, *PFC* prefrontal cortex

*Sburlea et al. 2017 [46] was included with their 2015 publication as they used the same data set in both studies.

**Table 6:** Quality assessment

*Quality assessment of controlled intervention studies*																
Paper	Q1	Q2	Q3	Q4	Q5	Q6	Q7	Q8	Q9	Q10	Q11	Q12	Q13	Q14	% Score	Overall Rating
(Calabro et al., 2017) [40]	1	CD	1	0	1	1	1	1	1	CD	1	0	1	1	10/14 = 71%	Good
(Chen et al., 2019) [41]	1	CD	1	0	1	1	NR	NR	1	NR	1	0	1	1	8/14 = 57%	Fair

*Quality assessment for observational cohort and cross-sectional studies*																
Paper	Q1	Q2	Q3	Q4	Q5	Q6	Q7	Q8	Q9	Q10	Q11	Q12	Q13	Q14	% Score	Overall Rating
(Al-Yahya et al., 2016 )[22]	1	0	CD	1	0	0	0	NA	1	NA	1	NA	NA	1	5/10 = 50%	Good
(Caliandro et al., 2020) [23]	1	0	CD	1	0	0	0	NA	1	NA	1	NA	NA	1	5/10 = 50%	Good
(Chang et al., 2019) [32]	1	0	CD	0	0	0	0	NA	1	NA	1	NA	NA	0	3/10 = 30%	Poor
(Chatterjee et al., 2019 )[33]	1	0	CD	1	0	0	0	1	1	NA	1	NA	NA	1	6/11 = 55%	Good
(Choi et al., 2016)[34]	1	0	CD	CD	0	0	0	NA	1	NA	1	NA	NA	1	4/10 = 40%	Fair
(García-Cossio et al., 2015) [35]	1	0	CD	CD	0	0	0	NA	1	NA	1	NA	NA	1	4/10 = 40%	Fair
(Hawkins et al., 2018) [36]	1	0	CD	1	0	0	0	NA	1	NA	1	NA	NA	1	5/10 = 50%	Good
(Hermand et al., 2019) [37]	1	0	CD	1	0	0	0	1	1	NA	1	NA	NA	1	6/11 = 55%	Good
(Lee et al., 2018) [30]	1	0	CD	CD	0	0	0	NA	1	NA	1	NA	NA	0	3/10 = 30%	Poor
(Liu et al., 2018) [38]	1	1	1	1	0	0	0	1	1	NA	1	NA	NA	1	8/11 = 73%	Good
(Mihara et al., 2007) [39]	1	0	CD	0	0	0	0	NA	0*	NA	1	NA	NA	0	2/10 = 20%	Fair
(Mitchell et al., 2018)[31]	1	0	CD	CD	0	0	0	NA	1	NA	1	NA	NA	1	4/10 = 40%	Fair
(Miyai et al., 2006) [24]	0	0	CD	0	0	0	0	NA	1	NA	1	NA	NA	0	2/10 = 20%	Poor
(Miyai et al., 2006) [25]	1	0	CD	0	0	0	0	NA	1	NA	1	NA	NA	1	4/10 = 40%	Fair
(Mori et al., 2018) [26]	1	0	CD	1	0	0	0	NA	1	NA	1	NA	NA	1	5/10 = 50%	Fair
(Saitou et al., 2000) [27]	1	0	CD	1	0	0	0	NA	0	NA	0	NA	NA	0	2/10 = 20%	Poor
(Sangani et al., 2015) [28]	1	0	CD	0	0	0	0	NA	1	NA	1	NA	NA	NA	3/9 = 33%	Poor
(Sburlea et al., 2015) [29]	1	0	CD	0	0	0	0	NA	0	1	1	NA	1	1	5/12 = 42%	Good

Questions were assessed based on the NIH Study Quality Assessment Tools (https://www.nhlbi.nih.gov/health-topics/study-quality-assessment-tools)

*CD* cannot determine, *NA* not applicable, 1: Yes, for the given question, 0: No, for the given question

*Independent measure (gait speed) was consistently applied within the stroke group but not between stroke and healthy older adults. Healthy older adults were asked to walk at their comfortable speed while stroke participants were asked to walk at their fast, comfortable speed

**Details obtained through Mitchell et al. 2019 [44]

## Abbreviations

[18F]-FDG-PET: Positron emission topography with fluorodeoxyglucose tracing
EEG: Electroencephalography
fMRI: Functional magnetic resonance imaging
fNIRS: Functional near-infrared spectroscopy
MeSH: Medical subject headings
NIH: National Institutes of Health
PET: Positron emission topography
PFC: Prefrontal cortex
PMC: Premotor cortex
SMA: Supplementary motor area
SMC: Sensorimotor cortex
SPECT: Single-photon emission computerized tomography

## References

[1] Lord SE, McPherson K, McNaughton HK, Rochester L, Weatherall M (2004). Community ambulation after stroke: how important and obtainable is it and what measures appear predictive?. Arch Phys Med Rehabil.

[2] Rudberg A, Berge E, Laska A, Jutterström S, Näsman P, Sunnerhagen KS (2020). Stroke survivors’ priorities for research related to life after stroke. Top Stroke Rehabil..

[3] Blennerhassett JM, Levy CE, Mackintosh A, Yong A, McGinley JL (2018). One-quarter of people leave inpatient stroke rehabilitation with physical capacity for community ambulation. J Stroke Cerebrovasc Dis.

[4] Perry J, Garrett M, Gronley JK, Mulroy SJ (1995). Classification of walking handicap in the stroke population. Stroke.

[5] da Silva MAS, Borich M (2019). Commentary on: increased sensorimotor cortex activation with decreased motor performance during functional upper extremity tasks poststroke. J Neurol Phys Ther.

[6] Boyd LA, Hayward KS, Ward NS, Stinear CM, Rosso C, Fisher RJ (2017). Biomarkers of stroke recovery: consensus-based core recommendations from the stroke recovery and rehabilitation roundtable. Int J Stroke.

[7] CIHR. Research in priority areas [Internet]. 2020. https://cihr-irsc.gc.ca/e/50077.html.

[8] Hamacher D, Herold F, Wiegel P, Hamacher D, Schega L (2015). Brain activity during walking: a systematic review. Neurosci Biobehav Rev.

[9] la Fougère C, Zwergal A, Rominger A, Förster S, Fesl G, Dieterich M (2010). Real versus imagined locomotion: a [18F]-FDG PET-fMRI comparison. Neuroimage.

[10] Suzuki M, Miyai I, Ono T, Kubota K (2008). Activities in the frontal cortex and gait performance are modulated by preparation. An fNIRS study. Neuroimage.

[11] Mirelman A, Maidan I, Bernad-Elazari H, Shustack S, Giladi N, Hausdorff JM (2017). Effects of aging on prefrontal brain activation during challenging walking conditions. Brain Cogn.

[12] Pelicioni PHS, Tijsma M, Lord SR, Menant J (2019). Prefrontal cortical activation measured by fNIRS during walking: effects of age, disease and secondary task. PeerJ.

[13] Vitorio R, Stuart S, Rochester L, Alcock L, Pantall A (2017). fNIRS response during walking—artefact or cortical activity? A systematic review. Neurosci Biobehav Rev.

[14] Holtzer R, Verghese J, Allali G, Izzetoglu M, Wang C, Mahoney JR (2016). Neurological gait abnormalities moderate the functional brain signature of the posture first hypothesis. Brain Topogr.

[15] Allali G, Blumen HM, Devanne H, Pirondini E, Delval A, Van De VD (2018). Brain imaging of locomotion in neurological conditions. Clin Neurophysiol.

[16] Gramigna V, Pellegrino G, Cerasa A, Cutini S, Vasta R, Olivadese G (2017). Near-infrared spectroscopy in gait disorders: is it time to begin?. Neurorehabil Neural Repair.

[17] Yang M, Yang Z, Yuan T, Feng W, Wang P (2019). A systemic review of functional near-infrared spectroscopy for stroke: current application and future directions. Front Neurol.

[18] Scholkmann F, Kleiser S, Metz AJ, Zimmermann R, Mata Pavia J, Wolf U (2014). A review on continuous wave functional near-infrared spectroscopy and imaging instrumentation and methodology. Neuroimage [Internet]..

[19] Thompson T, Steffert T, Ros T, Leach J, Gruzelier J (2008). EEG applications for sport and performance. Methods.

[20] Verger A, Guedj E (2018). The renaissance of functional 18F-FDG PET brain activation imaging. Eur J Nucl Med Mol Imaging..

[21] National Institutes of Health. Quality Assessment Tools [Internet]. 2014. https://www.nhlbi.nih.gov/health-topics/study-quality-assessment-tools

[22] Al-Yahya E, Johansen-Berg H, Kischka U, Zarei M, Cockburn J, Dawes H (2016). Prefrontal cortex activation while walking under dual-task conditions in stroke: a multimodal imaging study. Neurorehabil Neural Repair.

[23] Caliandro P, Molteni F, Simbolotti C, Guanziroli E, Iacovelli C, Reale G (2020). Exoskeleton-assisted gait in chronic stroke: an EMG and functional near-infrared spectroscopy study of muscle activation patterns and prefrontal cortex activity. Clin Neurophysiol.

[24] Miyai I, Yagura H, Oda I, Konishi I, Eda H, Suzuki T (2002). Premotor cortex is involved in restoration of gait in stroke. Ann Neurol.

[25] Miyai I, Suzuki M, Hatakenaka M, Kubota K (2006). Effect of body weight support on cortical activation during gait in patients with stroke. Exp Brain Res.

[26] Mori T, Takeuchi N, Izumi S-I (2018). Prefrontal cortex activation during a dual task in patients with stroke. Gait Posture Elsevier.

[27] Saitou H, Yanagi H, Hara S, Tsuchiya S, Tomura S (2000). Cerebral blood volume and oxygenation among poststroke hemiplegic patients: effects of 13 rehabilitation tasks measured by near-infrared spectroscopy. Arch Phys Med Rehabil.

[28] Sangani S, Lamontagne A, Fung J (2015). Cortical mechanisms underlying sensorimotor enhancement promoted by walking with haptic inputs in a virtual environment. Prog Brain Res..

[29] Sburlea AI, Montesano L, Cano-De La Cuerda R, Alguacil Diego IM, Miangolarra-Page JC, Minguez J (2015). Detecting intention to walk in stroke patients from pre-movement EEG correlates. J Neuroeng Rehabil..

[30] Lee S, Lee H, Kim D, Chang W, Choi B, Ryu G, et al. Wearable hip-assist robot modulate cortical activation during gait in stroke patients. Eur Stroke J. 2018;3(1S):126. 10.1186/s12984-020-00777-0 PMC7596937 33121535

[31] Mitchell T, Starrs F, Thiel A, Paquette C. Changes in complex locomotor control in chronic stroke. Int J Stroke. 2018;13(2S):43–4.

[32] Chang WC, Ko LW, Yu KH, Ho YC, Chen CH, Jong YJ (2019). EEG analysis of mixed-reality music rehabilitation system for post-stroke lower limb therapy. J Soc Inf Disp.

[33] Chatterjee SA, Fox EJ, Daly JJ, Rose DK, Wu SS, Christou EA (2019). Interpreting prefrontal recruitment during walking after stroke: Influence of individual differences in mobility and cognitive function. Front Hum Neurosci.

[34] Choi J, Kang H, Chung SH, Kim Y, Lee UH, Lee JM, et al. Detecting voluntary gait intention of chronic stroke patients towards top-down gait rehabilitation using EEG. IEEE. 2016. p. 1560–3. 10.1109/EMBC.2016.7591009 28268625

[35] García-Cossio E, Severens M, Nienhuis B, Duysens J, Desain P, Keijsers N (2015). Decoding sensorimotor rhythms during robotic-assisted treadmill walking for brain computer interface (BCI) applications. PLoS ONE.

[36] Hawkins KA, Fox EJ, Daly JJ, Rose DK, Christou EA, McGuirk TE (2018). Prefrontal over-activation during walking in people with mobility deficits: interpretation and functional implications. Hum Mov Sci.

[37] Hermand E, Tapie B, Dupuy O, Fraser S, Compagnat M, Salle JY (2019). Prefrontal cortex activation during dual task with increasing cognitive load in subacute stroke patients: a pilot study. Front Aging Neurosci.

[38] Liu YC, Yang YR, Tsai YA, Wang RY, Lu CF (2018). Brain activation and gait alteration during cognitive and motor dual task walking in stroke—a functional near-infrared spectroscopy study. IEEE Trans Neural Syst Rehabil Eng.

[39] Mihara M, Miyai I, Hatakenaka M, Kubota K, Sakoda S (2007). Sustained prefrontal activation during ataxic gait: a compensatory mechanism for ataxic stroke?. Neuroimage.

[40] Calabro RS, Naro A, Russo M, Leo A, De Luca R, Balletta T (2017). The role of virtual reality in improving motor performance as revealed by EEG: a randomized clinical trial. J Neuroeng Rehabil.

[41] Chen I-H, Yang Y-R, Lu C-F, Wang R-Y (2019). Novel gait training alters functional brain connectivity during walking in chronic stroke patients: a randomized controlled pilot trial. J Neuroeng Rehabil.

[42] Contreras-Vidal JL, Bortole M, Zhu F, Nathan K, Venkatakrishnan A, Francisco GE (2018). Neural decoding of robot-assisted gait during rehabilitation after stroke. Am J Phys Med Rehabil.

[43] Miyai I, Yagura H, Hatakenaka M, Oda I, Konishi I, Kubota K (2003). Longitudinal optical imaging study for locomotor recovery after stroke. Stroke.

[44] Mitchell T, Starrs F, Thiel A, Paquette C (2019). Impaired sensorimotor processing during complex gait precedes behavioral changes in middle-aged adults. J Gerontol Biol Sci.

[45] Lee SH, Lee HJ, Shim Y (2020). Wearable hip-assist robot modulates cortical activation during gait in stroke patients: afunctional near-infrared spectroscopy study. J NeuroEngineering Rehabil.

[46] Sburlea AI, Montesano L, Minguez J. Advantages of EEG phase patterns for the detection of gait intention in healthy and stroke subjects. J Neural Eng. 2017;14:036004. 10.1088/1741-2552/aa5f2f 28291737

[47] Jurcak V, Tsuzuki D, Dan I (2007). 10/20, 10/10, and 10/5 systems revisited: their validity as relative head-surface-based positioning systems. Neuroimage.

[48] Clark DJ, Rose DK, Ring SA, Porges EC (2014). Utilization of central nervous system resources for preparation and performance of complex walking tasks in older adults. Front Aging Neurosci.

[49] Bradnam LV, Stinear CM, Barber PA, Byblow WD (2012). Contralesional hemisphere control of the proximal paretic upper limb following stroke. Cereb Cortex.

[50] Carey LM, Abbott DF, Egan GF, Bernhardt J, Donnan GA (2005). Motor impairment and recovery in the upper limb after stroke: behavioral and neuroanatomical correlates. Stroke.

[51] Dodd KC, Nair VA, Prabhakaran V (2017). Role of the contralesional vs ipsilesional hemisphere in stroke recovery. Front Hum Neurosci..

[52] Lee JH, Kyeong S, Kang H, Kim DH (2019). Structural and functional connectivity correlates with motor impairment in chronic supratentorial stroke: a multimodal magnetic resonance imaging study. NeuroReport.

[53] Rehme AK, Eickhoff SB, Rottschy C, Fink GR, Grefkes C (2012). Activation likelihood estimation meta-analysis of motor-related neural activity after stroke. Neuroimage.

[54] Luft AR, Forrester L, Macko RF, McCombe-Waller S, Whitall J, Villagra F (2005). Brain activation of lower extremity movement in chronically impaired stroke survivors. Neuroimage.

[55] Enzinger C, Johansen-Berg H, Dawes H, Bogdanovic M, Collett J, Guy C (2008). Functional MRI correlates of lower limb function in stroke victims with gait impairment. Stroke.

[56] Di Pino G, Pellegrino G, Assenza G, Capone F, Ferreri F, Formica D (2014). Modulation of brain plasticity in stroke: a novel model for neurorehabilitation. Nat Rev Neurol.

[57] Peters S, Ivanova TD, Lakhani B, Boyd LA, Staines WR, Handy TC (2018). Symmetry of cortical planning for initiating stepping in sub-acute stroke. Clin Neurophysiol.

[58] Wilson J, Allcock L, Mc Ardle R, Taylor J-P, Rochester L (2019). The neural correlates of discrete gait characteristics in ageing: a structured review. Neurosci Biobehav Rev.

[59] Maidan I, Bernad-Elazari H, Gazit E, Giladi N, Hausdorff JM, Mirelman A (2015). Changes in oxygenated hemoglobin link freezing of gait to frontal activation in patients with Parkinson disease: an fNIRS study of transient motor-cognitive failures. J Neurol.

[60] Colcombe SJ, Kramer AF, Erickson KI, Scalf P (2005). The implications of cortical recruitment and brain morphology for individual differences in inhibitory function in aging humans. Psychol Aging.

[61] Harada T, Miyai I, Suzuki M, Kubota K (2009). Gait capacity affects cortical activation patterns related to speed control in the elderly. Exp Brain Res.

